# Geosocial Networking Apps Use Among Sexual Minority Men in Ecuador: An Exploratory Study

**DOI:** 10.1007/s10508-021-01921-0

**Published:** 2021-06-11

**Authors:** Carlos Hermosa-Bosano, Paula Hidalgo-Andrade, Clara Paz

**Affiliations:** School of Psychology, Universidad de Las Américas (UDLA), Redondel del Ciclista, Antigua Vía a Nayón, Campus UDLAPark, 170124 Quito, Ecuador

**Keywords:** Geosocial networking, Mobile apps, Gay dating apps, Men who have sex with men, Sexual orientation

## Abstract

Geosocial networking applications (GSN apps) have become important socialization contexts for sexual minority men (SMM). Despite their popularity, there is limited research carried out in Latin American countries and no single previous study done in Ecuador. To fill this gap, this exploratory study described and analyzed the relationships between the sociodemographic characteristics of SMM using GSN apps, their sought and fulfilled expectations, profile shared and sought characteristics, and the evaluation of their experiences as users including their perceptions of support, and discrimination. We used an online recruited sample of 303 participants enrolled between November 2019 and January 2020. Most respondents used Grindr and reported spending up to 3 h per day using apps. Most common sought expectations were getting distracted, meeting new friends, and meeting people for sexual encounters. The least met expectation was meeting someone to build a romantic relationship with. When asked about their profiles, participants reported sharing mainly their age, photographs, and sexual role. Participants also prioritized these characteristics when looking at others’ profiles. When asked about their experiences, most reported having been discriminated against, weight being the main reason for it. Some participants also indicated having received emotional support from other users. Correlation analyses indicated significant but weak relationships among the variables. Results indicated a positive correlation between time as an active GSN app user and higher experiences of discrimination. Likewise, higher number of used apps related positively with levels of received support. These results provide information that could inform future research in the country and the region regarding GSN apps use among SMM, socialization practices, and modern dating tools.

## Introduction

Among the countries in Latin America, Ecuador stands as one of the most conservative (Moncagatta et al., 2020). According to the Ecuadorian Statistical and Census Institute (INEC, in Spanish), 91.9% of the population in the country reports having a religion (INEC, 2012). The place religion occupies in the life of its citizens, as well as other cultural factors such as Machismo and Familism, may explain why Ecuador is one of the least accepting countries toward homosexuality in the region, next to Bolivia and Peru (Pew Research Center, 2013; Tummino & Bintrim, 2016). Though there have been significant advancements in terms of legal protections, there are still reports of high levels of discrimination against gay, bisexual, and other sexual minority men (SMM) in settings such as the education, labor, and health systems (INEC, 2013).

Recently, in 2017, the National Research Institute of Public Health (INSPI, in Spanish) and Corporación Kimirina collected data of 748 SMM in Quito and Guayaquil and reported that gay bars and parks were the places where men most frequently meet (INSPI & Corporación Kimirina, 2017). This information allows researchers to understand the physical and social characteristics and dynamics of the spaces where members of the queer community navigate. However, to date, there has been no research conducted to learn about SMM use of online socialization contexts such as Geosocial Networking Applications (GSN apps).

With the invention of smartphones and app-based operating systems, GSN apps and other social media have become essential settings for queer communities worldwide (Grosskopf et al., 2014; Wang et al., 2018; Zou & Fan, 2017). In Ecuador, previous national data has indicated that approximately 63.2% and 70.2% of 16-to-34 year-olds have a smartphone (INEC, 2018). Thus, the higher access to digital devices using GPS technologies may translate into higher use of apps among SMM. Considering the lack of information on this subject in the country, we conducted an exploratory, quantitative study intended to describe the characteristics of GSN apps users, their expectations sought and fulfilled when using GSN apps, the characteristics that they share on their profiles and the ones that they look for when examining others’ profiles, their evaluation of their experiences as well as social support, and discrimination events. We also explored the relationships between sociodemographic characteristics and the previously mentioned variables.

### Geosocial Networking Apps

The Internet has long been used by SMM around the globe to meet other men, express their sexuality, and build online and offline communities (Grosskopf et al., 2014; Rosser et al., 2011). Previous studies in the U.S. estimate that up to 85% of SMM have used the Internet to meet other people, and “as many as 94% […] report ever having had sex with a partner met online” (Rosser et al., 2011, S92). SMM-specific dating apps such as Grindr, Hornet, and Scruff, as well as other non-SMM-specific apps like Tinder, are among the most popular venues used by SMM (Badal et al., 2018; Rice et al., 2012). However, other social media such as Facebook and Instagram have become notable means for social and sexual socialization among SMM. Despite not being specifically designed for dating, these contexts have become platforms for many men to live their identity in a public manner, leading to the formation of social support systems around them (Rubin & McClelland, 2015).

There are several reasons why these apps are widely popular. First, they are easily accessible, free to download, and can be used on portable devices such as smartphones and tablets. Additionally, GSN apps enable users to connect others by using Global Positioning System (GPS) technologies. This feature allows people to identify nearby or geographically distant users who could become potential friends, as well as sexual or romantic partners (Ko et al., 2016; Zervoulis et al., 2020). This convenient and easy way to locate other people can bring additional benefits such as privacy and anonymity, especially for men fearing stigma (Rosser et al., 2011).

Since the launch of Grindr in 2009, studies on GSN apps among SMM have significantly expanded. Due to these apps’ potential to increase users’ social and sexual networks, most of the studies have analyzed the perceived risk and prevalence of HIV and other sexually transmitted infections (STIs; Wei et al., 2019), SMM’s involvement in sexual risk behaviors (i.e., condomless sex; group sexual activity; drug use; Grosskopf et al., 2014; Phillips et al., 2014; Winetrobe et al., 2014), as well as health-related practices (i.e., HIV/STIs testing; Holloway et al., 2014). Other studies have investigated mental health variables such as internalized homonegativity (Grosskopf et al., 2014), individual well-being (Zervoulis et al., 2020), among others. Furthermore, studies have discussed apps’ potentials to become intervention sites, as well as the effectiveness of different HIV/STIs prevention initiatives tailored for these populations (Cao et al., 2017; Contesse et al., 2020; Hightow-Weidman et al., 2015; Lee et al., 2018; Muessig et al., 2015).

Altogether, this research has provided information about the positive effects of app use as well as their negative toll on their users. Unfortunately, as Miller (2015b) has suggested, “much on the literature on Grindr and like applications stems from the perspective of analyzing sexual behavior, the outcome, rather than the motivations for using social networking applications in the first place” (p. 477). For this reason, we believe it is important to analyze what SMM expect to obtain when using online socialization platforms.

### Sought and Fulfilled Expectations

A framework to study app users’ expectations behind their media usage has been the Uses and Gratifications Model (Gerlich et al., 2015; Ruggiero, 2020). This conceptual framework was developed to understand how different media (e.g., television, internet, social networking sites) satisfy people’s social and psychological needs and desires (Ruggiero, 2020). According to this model, people use different types of technologies to obtain gratifications such as leisure, information, social connections, among others. When initial expectations are met, people obtain gratifications which lead to behavior change, impacting other outcomes such as media exposure and engagement.

In recent years, with the increased availability of online networking sites directed at SMM, studies have gathered information about the uses and gratifications of sexual minority users (Gudelunas, 2012; Miller, 2015b; Van De Wiele & Tong, 2014). Studies in this area suggest that SMM frequently use online sites to obtain sexual gratifications (Goedel & Duncan, 2015). In a quantitative study carried out with a randomly selected sample of Grindr users, Rice et al. (2012) found that 65.1% of their participants used the app to meet sexual partners. Similarly, Macapagal et al. (2018) found that 42.7% of the men in their sample reported seeking cybersex, and 68.9% to have physical sex with other men. These results highlight what Gudelunas (2012) suggests in terms of the construction of sexual capital. Based on Bourdieu’s concept of social capital, Gudelunas (2012) argued that SMM-specific sites allow users to build sexual capital by facilitating encounters among users and allow them to maintain connections with past and present sexual partners. However, as this author suggests, it is not only SMM-sites that allow this. Facebook, Tinder, and other apps such as Instagram, may serve to connect gay men and allow them to interact in sexual ways. In addition, non-specific SMM media such as Facebook can be used as extensions of gay sites to cross-reference the information of potential partners, obtain further data from more “credible” sources, and analyze information such as mutual friends to decide whether to engage or not in future interactions (Gudelunas, 2012).

Other gratifications are equally important. Studies have found that SMM often seek to explore different aspects of their identity by reaching other men, allowing them to express and live their sexuality in relatively safe and anonymous contexts (Goedel et al., 2017; Zervoulis et al., 2020). Connecting to other people perceived as having similar interests can facilitate self-acceptance processes and integrate them into a community that teaches them about the different aspects of having a queer identity (Zervoulis et al., 2020). Online communities can also offer spaces for people to facilitate coming out processes and expand a persons' connections, thereby, increasing their sense of well-being (Harper et al., 2016). This ability to express freely in online spaces might be especially relevant for SMM living in real-life hostile environments in which being openly gay is not an option (Zervoulis et al., 2020). It has been argued that apps can allow people to access places where they can feel safe without having to expose their identity (Miller, 2015b). It should be noted that these benefits might be limited based on location, since a limited number of connections, such as those in rural areas, may narrow the possibilities to create a sense of community and the amount of social support sources (Zervoulis et al., 2020).

### Shared and Sought Profile Characteristics

An important aspect of GSN apps use is the creation of a personal profile. Personal advertisements allow people to share their personal characteristics as a way to attract others, state what they search for in a partner, and express what they are willing to do with others (e.g., meet them in person, engage in cybersex; Miller, 2015a). This information also acts as the basic resource for users to filter among hundreds of potential partners. Previous research has found that gay men share more physical characteristics in their profiles than lesbians, who usually highlight their personality traits (Miller, 2015a). This emphasis on aesthetics leads men to share photographs accentuating their entire bodies or certain body parts (e.g., chest, arms), as well as information about their body type, height, and weight (Miller, 2019). These practices are often associated with ideas about masculinity and femininity, and through them, power, control, and desirability (Miller, 2015a, 2019). Similarly, self-labels based on sexual roles (i.e., top, bottom, versatile) are characteristics often shared in profiles. Like body aesthetics, sexual preferences during anal intercourse are usually associated with social constructions about gender roles (Moskowitz et al., 2008; Moskowitz & Roloff, 2017). Thus, those who share being a top are perceived as more masculine, dominant, and controlling in both the sexual and relational arenas; whereas bottoms tend to be perceived as less masculine, more submissive, and more emotionally attached (Moskowitz & Garcia, 2019).

### User Experiences and Discrimination

Profile-based filtering, as well as the quality of interactions between SMM, can lead some men to have negative experiences when using apps. In Miller’s (2015b) study, most participants reported having experienced negative feelings after using SMM-specific social networks. Participants in this study reported having felt loneliness, anxiety, shame, among others. Also, there has been reports of discrimination related to femmephobia, fatphobia, ageism, and racism. In a critical essay written by Conte (2018), Grindr was described as a space plagued with hegemonic narratives that celebrate whiteness, masculinity, and muscularity. These sociocultural ideologies reproduced in profiles and conversations, often exclude queer identities and bodies thus creating a double marginalization: the one created by the straight community, and the one coming from inside the queer community (Conte, 2018). Altogether, these experiences can have negative effects on users’ well-being, leading some men to experience internalized homophobia and lowered self-esteem (Miller & Behm-Morawitz, 2016).

### Research in Latin America

To date, much of the research on GSN apps use has been primarily conducted in the Global North. In Latin America, there has been some research published in Brazil (Morelli & Pereira, 2018; Queiroz et al., 2019) and Peru (Chow et al., 2017). Queiroz et al. (2019), for example, published a study based on a Facebook-recruited sample of 2,250 men in Brazil and found that Tinder and Grindr were the most used applications. Similarly, in Peru, Chow et al. (2017) performed a study analyzing data from 312 SMM and 89 transgender women and found that Facebook and the mobile version of Manhunt were the most used apps in their sample. Altogether, this data reveals that preferred apps may change across countries. It should be noted though, that both studies were interested in analyzing sexual health variables such as HIV and STIs prevalence among app users, leaving behind the analysis of other aspects such as users’ motives for using apps, their experiences, and the social support obtained from app-met partners.

Based on these observations, and considering the lack of information in a country like Ecuador, we carried out a study aimed to (1) describe the sociodemographic characteristics of SMM using GSN apps, (2) analyze the expectations sought and fulfilled among the men in the sample, (3) analyze profile shared and sought characteristics, and (4) describe the experiences of SMM including the received social support and discrimination from other app users. We were also interested in (5) analyzing the relationships between sociodemographic characteristics and the previously mentioned variables. We believe the obtained results, though exploratory in nature, provide valuable information on app use in a country where research on these matters is still scarce.

## Method

### Participants and Procedure

This was an exploratory, cross-sectional study that used an online recruited sample of SMM that reported being GSN app users. To be eligible, participants needed to reside in Ecuador, be 18 years or older, self-identify as male, report having used GSN apps in the past year, and report having at least one male sexual partner in the year before the survey. We collected the data analyzed for this article between December 2019 and February 2020 through an online survey on SurveyMonkey (SurveyMonkey Inc., 2020) distributed on social media (Facebook, Instagram, Twitter, WhatsApp) using snowball sampling. We sent an invitation to personal friends, colleagues, and acquaintances who we knew were part of the population of interest to act as seeds of the snowball sample. We asked them to participate and re-send the survey to their contacts. Also, we posted the study invitation on the Facebook page of Universidad de Las Américas’ Psychology Department and that of *Grupo Cerebro, Emoción y Conducta*, the research group to which the authors belong. This invitation included an image designed for the study, the eligibility criteria, and the survey link. We also printed flyers and left them in different areas of the Psychology Department, and other university facilities. The print version of the invitation included a QR code to facilitate access to the survey.

Self-selected participants were instructed to complete the survey which included filter questions based on eligibility criteria. For example, if a participant reported not having used GSN apps in the last year, the survey automatically ended. When entering the survey, participants had to read an informed consent page explaining the study objectives, procedures, data management protocol, as well as the potential harms and benefits. They did not receive incentives to participate. The survey was conducted in Spanish (Ecuador’s official language), was anonymous, and confidential. Participants could exit the survey at any point. The questionnaire took approximately 10 min to be completed. IRB approval was granted to the corresponding author before launching the survey (CEISH-USFQ code: 2019-109E).

### Measures

The research team designed the survey for this study. To ensure validity and context sensibility, the initial instrument was reviewed by four national and two international academic experts in the fields of sexuality, sexual health and gender issues. Their main suggestions included reducing the number of items and sections. The revised version of the survey was tested with five app users to gather qualitative evidence on the survey duration and comprehension. They were acquaintances of the first author, met the eligibility criteria for the study, and did not participate in the final study. The final instrument is available upon request to the corresponding author. Sections analyzed in this article include the following:

#### Demographic Information

Sociodemographic characteristics were first presented and included items on sex assigned at birth (male, female, intersex), self-reported gender (male, female, transsexual, transgender), age, sexual orientation, the gender of sexual partners in the past year, and relationship status. Other questions assessed self-reported ethnicity, nationality, country of residence, city of residence, education level, religious practice, occupation, and monthly income (measured in USD, the local currency).

#### Characteristics of App Use

We included multiple-choice questions to assess the amount of time participants were active users on GSN apps (1 = less than 6 months, 2 = between six and 11 months, 3 = between 1 and 2 years, 4 = more than 2 years), GSN apps used during the past year, and the amount of time spent per day (1 = less than an hour, 2 = between 1 and 3 h, 3 = more than 3 h). The list of apps included Tinder, Happn, Grindr, Scruff, Hornet, Manhunt, Daddyhunt, Facebook, and Instagram. Participants could check all the answers that applied, and if they used other apps, they were given the possibility to identify their name(s).

#### Sought and Fulfilled Expectations

We explored participants' expectations using select-all-that-apply items that represented eight different expectations: making new friends, having a romantic and stable relationship, having sexual experiences, looking for tourist information when traveling, forgetting an ex, connecting with people with the same sexual orientation, looking for support or company, and getting distracted. The items included in this section were inspired by the 13 factors explored in the Tinder Motives Scale (TMS), a 58-item questionnaire created by Timmermans and De Caluwé (2017) based on the Uses and Gratification Model. Original factors in the TMS include social approval, relationship seeking, sexual experience, flirting/social skills, travelling, forgetting an ex, belongingness, peer pressure, socializing, sexual orientation, pass time/entertainment, distraction, and curiosity. In our questionnaire, we asked participants to determine whether the item expressed their expectation or not (i.e., sought gratification, non-sought gratification), and whether their expectations had been met or not (i.e., fulfilled gratification, non-fulfilled gratification).

#### Shared and Sought Profile Characteristics

In this section, we formulated multiple-choice questions to assess the profile characteristics users shared in their main app profile. Participants were also asked to identify the elements that they noticed while viewing other users’ profiles. Options included: age, gender identity, occupation, real name, nickname, links to other social media profiles (e.g., Facebook, Instagram), geographical location, photograph, phone number, height, weight/body type, personal description, relationship status, sexual role (e.g., top, bottom, versatile), sexual preferences, motives for meeting others (e.g., friendship, sexual encounter), and penis size. We also explored the self-reported characteristics of profile pictures. Participants had to identify whether their profile picture had only their face, only their body, had both their face and body, was a real picture with other people, was a fake picture, was a picture of something important to them (e.g., a pet, a place, a logo), was an irrelevant picture, or whether their profile did not have a photograph.

#### User Experiences, Social Support, and Discrimination

We asked participants to evaluate their overall experience with GSN dating apps. This question had a five-point Likert response scale that ranged from 1 = very negative to 5 = very positive. We also asked participants to respond in a Likert-scale if they had received social support from people met through apps. Options were: 1 = never, 2 = seldom, 3 = sometimes, 4 = frequently, and 5 = always. We included a select-all-that-apply question related to the type of received social support: (1) emotional support, (2) advice and recommendations to solve problems, (3) constructive criticism, and (4) instrumental support. In addition, we added a question to evaluate the importance of received support. This item had a five-point Likert-type response that ranged from 1 = not important to 5 = very important. Finally, participants were asked to identify through a yes/no question whether they had been subjected to any sort of aggression by other app users (i.e., insults, threats). Participants had to identify the reason for being discriminated against. Options included sexual orientation, weight/body type, gender expression, skin color, ethnicity, beliefs, nationality, and having an STI. Finally, as part of the analysis of negative experiences, we included a yes/no question to evaluate whether the participant knew if someone had created a fake profile using their photographs and/or their personal information (i.e., catfishing).

### Statistical Analyses

We used descriptive statistics to identify the characteristics of the responses. Pearson’s correlation and one-way ANOVAs were used to analyze relations between continuous (i.e., age, monthly income) and categorical sociodemographic variables (i.e., sexual orientation, ethnicity, relationship status, nationality, country of residence, education level, religious practice) with app-use related variables (i.e., number of used GSN apps, daily amount of time using GSN apps, number of fulfilled expectations, number of unfulfilled expectations, number of profile shared contents, number of searched contents, rate of overall experience, frequency of received support, importance of received support, discrimination experience, catfishing experience) accordingly. All tests were two-tailed, and the significance level was set to *p* < .05. All the analyses were performed using SPSS 25. Only significant results were reported.

## Results

### Sample Characteristics

A total of 456 subjects accessed the online survey, and 303 responses were analyzed after meeting the inclusion criteria. In total, 153 participants (33.5%) were excluded due to not meeting eligibility criteria such as being an adult SMM, being a GSN apps user, having had sex with another male in the past year, and quitting the survey before completion. Eligible participants ranged from 18 to 62 years, with a mean age of 26.5 years (SD = 6.5). Most of the participants, 263 (86.8%), reported having sex only with men during the year before taking the survey. The majority reported being gay (78.5%, *n* = 238) and being single (90.8%, *n* = 275). Table 1 shows the sample’s sociodemographic composition in further detail.

**Table 1:** Sociodemographic characteristics of the sample

	*n* (%)
Age (*n* = 298; *M* = 26.5; SD = 6.5)	
18–24	136 (44.9)
25–34	127 (41.9)
35–44	28 (9.2)
45–64	7 (2.3)
Missing data	5 (1.7)
Sex at birth (*n* = 303)	
Male	301 (99.3)
Female	1 (0.3)
Intersex	1 (0.3)
Self-identified gender (*n* = 303)	
Male	302 (99.7)
Transgender	1 (0.3)
Sexual orientation (*n* = 303)	
Gay	238 (78.5)
Bisexual	65 (21.5)
Past year sexual partners’ gender (*n* = 303)	
Only men	263 (86.8)
Both men and women	40 (13.2)
Ethnicity (*n* = 303)	
Multiracial (*Mestizo*)	254 (83.8)
White	25 (8.3)
Indigenous	8 (2.6)
Montubio (*Mestizo*-Coastal region)	7 (2.3)
Black (Afro-Ecuadorian)	5 (1.7)
Other	2 (0.7)
None	2 (0.7)
Nationality (*n* = 303)	
Ecuadorian	277 (91.4)
Other	26 (8.6)
Highest education completed (*n* = 303)	
Primary school	1 (0.3)
High school	107 (35.3)
Technical degree	39 (12.9)
Undergraduate degree	114 (37.6)
Graduate degree or higher	42 (13.9)
Monthly income (USD) (*n* = 303)	
Less than 394 USD (Minimum wage)	127 (41.9)
395–1182 USD	134 (44.2)
More than 1183 USD (More than 3 minimum wages)	42 (13.9)
Marital status (*n* = 303)	
Single	275 (90.8)
Cohabitation	19 (6.3)
Civil union	4 (1.3)
Married	2 (0.7)
Separated	2 (0.7)
Divorced	1 (0.3)
Relationship status (*n* = 303)	
In a stable relationship, without cohabitation	26 (8.6)
In a stable relationship, with cohabitation	39 (12.9)
Currently not in a relationship	238 (78.5)
Practicing a religion (*n* = 303)	
Yes	122 (40.3)
No	131 (43.2)
Does not have a religion	50 (16.5)

### Characteristics of Apps Use

Table 2 details app use among respondents. The sample was heterogeneous regarding the amount of time as an active app user; 33.3% (*n* = 101) reported having used apps for less than 6 months, 16.8% (*n* = 51) between 6 and 11 months, 19.1% (*n* = 58) between 1 and 2 years, and 30.7% (*n* = 93) more than 2 years. Regarding the number of apps used, people reported using between one and nine apps, with a mean of 2.97 apps (SD = 1.4). In addition, 49.5% (*n* = 150) reported using them less than an hour per day, 35.3% (*n* = 107) between 1 and 3 h per day, and 15.2% (*n* = 46) more than 3 h per day. Analyses revealed that the most used apps were Grindr (87.5%, *n* = 265) followed by Tinder (64.7%, *n* = 196), Facebook (60.7%, *n* = 184), and Instagram (49.8%, *n* = 151).

**Table 2:** Characteristics of app use in the sample

	*n* (%)
Apps used (*n* = 303; *M* = 2.97; SD = 1.4)	
Grindr	265 (87.5)
Tinder	196 (64.7)
Facebook	184 (60.7)
Instagram	151 (49.8)
Scruff	39 (12.9)
Manhunt	20 (6.6)
Hornet	18 (5.9)
Other (Badoo, Blued, Howlr, Lp, Moovz, Taimi, Twitter, WhatsApp, Messenger)	19 (6.3)
Happn	3 (1.0)
Daddyhunt	3 (1.0)
Time as an active app user (*n* = 303)	
Less than 6 months	101 (33.3)
Between 6 and 11 months	51 (16.8)
Between 1 year and 2 years	58 (19.1)
More than 2 years	93 (30.7)
Spent time on apps per day (*n* = 303; *M* = 1.67; SD = 0.7)	
Less than an hour per day	150 (49.5)
Between 1 and 3 h per day	107 (35.3)
More than 3 h per day	46 (15.2)
Number of fulfilled expectations (*n* = 303; *M* = 4.26; SD = 1.9)	
Distraction	259 (85.5)
Connect with people with my same sexual orientation	237 (78.2)
Have new sexual experiences	220 (72.6)
Make new friends	175 (57.8)
Look for touristic information when traveling	147 (48.5)
Have a stable romantic relationship	75 (24.8)
Look for support or company	108 (35.6)
Forget my ex-partner	69 (22.8)
Number of unfulfilled expectations (*n* = 303; *M* = 1.46; SD = 1.6)	
Distraction	19 (6.3)
Connect with people with my same sexual orientation	25 (8.3)
Have new sexual experiences	23 (7.6)
Make new friends	67 (22.1)
Look for touristic information when traveling	55 (18.2)
Have a stable romantic relationship	143 (47.2)
Look for support or company	53 (17.5)
Forget my ex-partner	56 (18.5)
Number of shared characteristics (*n* = 303; *M* = 8.00; SD = 3.7)	
Age	279 (92.1)
Photograph	235 (77.6)
Gender identity	199 (65.7)
Sexual role (e.g., top, bottom, versatile)	188 (62.0)
Height	182 (60.1)
Motives for meeting others (e.g., friendship, sexual encounters)	180 (59.4)
Weight/body type	137 (45.2)
Real name	134 (44.2)
Geographic location	122 (40.3)
Relationship status	112 (37.0)
Personal description	111 (36.6)
Sexual preferences	108 (35.6)
Links to other social media profiles (e.g., Facebook, Instagram)	104 (34.3)
Nickname	91 (30.0)
Phone number	77 (25.4)
Occupation	69 (22.8)
Penis size	21 (6.9)
Number of sought characteristics (*n* = 303; *M* = 6.30; SD = 3.5)	
Age	243 (80.0)
Photograph	233 (76.9)
Gender identity	98 (32.3)
Sexual role (e.g., top, bottom, versatile)	188 (62.0)
Height	115 (38.0)
Motives for meeting others (e.g., friendship, sexual encounters)	132 (43.6)
Weight/body type	98 (32.3)
Real name	90 (29.7)
Geographic location	91 (30.0)
Relationship status	80 (26.4)
Personal description	112 (37.0)
Sexual preferences	77 (25.4)
Links to other social media profiles (e.g., Facebook, Instagram)	85 (28.1)
Nickname	18 (5.9)
Phone number	35 (11.6)
Occupation	63 (20.8)
Penis size	30 (9.9)
Photograph characteristics (*n* = 303)	
Only face	134 (44.2)
Both face and body	90 (29.7)
Only body	33 (10.9)
Irrelevant picture	19 (6.3)
Picture of something important (e.g., pet, place, logo)	9 (3.0)
Real picture with other people	3 (1.0)
Fake picture	2 (0.7)
No photograph	13 (4.3)

### Sought and Fulfilled Expectations

Participants’ most reported expectations were getting distracted (91.8%, *n* = 278), connecting with people with the same sexual orientation (86.5%, *n* = 262), having new sexual experiences (80.2%, *n* = 243), making new friends (79.9%, *n* = 242), and having a stable and romantic relationship (72%, *n* = 218). Most of the sample (98.7%, *n* = 299) reported that at least one of their sought expectations was fulfilled (Table 2). The least met expectation was meeting someone to have a stable and romantic relationship (47.2%, *n* = 143).

### Shared and Sought Profile Characteristics

Table 2 presents the characteristics that participants reported sharing in their profile. The five most frequently shared characteristics on participants’ profiles were their age (92.1%, *n* = 279), photograph (77.6%, *n* = 235), gender identity (65.7%, *n* = 199), sexual role (62.0%, *n* = 188), and height (60.1%, *n* = 182). The characteristics often sought in other users’ profiles included their age (80.0%, *n* = 243), photograph (76.9%, *n* = 233), sexual role (62.0%, *n* = 188), the motives for meeting others in the app (43.6%, *n* = 132), and their height (38.0%, *n* = 115).

Regarding the characteristics of participants’ main profile picture, 4.3% (*n* = 13) reported not using any kind of picture. Of those who did (95.7%, *n* = 290), 44.2% (*n* = 134) indicated their photograph had only their face, 29.7% (*n* = 90) had both their face and body, 10.9% (*n* = 33) had a picture of only their body, 6.3% (*n* = 19) had an irrelevant picture, 3.0% (*n* = 9) had a picture of something important to them, 1.0% (*n* = 3) was a picture of him with other people, and 0.7% (*n* = 2) had a fake profile picture.

### User Experiences, Social Support, and Discrimination

Table 3 details information about participants’ experiences using apps. Participants varied on the ratings of their overall experience as GSN apps users. 15.5% (*n* = 47) reported that their experience had been very or somewhat negative, 47.9% (*n* = 145) rated their experience as neutral (i.e., neither negative nor positive), and 36.6% (*n* = 111) reported that their experience had been very or somewhat positive.

**Table 3:** Participants’ experiences using apps

	*n* (%)
Overall experience rating (*n* = 303; *M* = 3.22; SD = 0.9)	
Very negative	13 (4.3)
Somewhat negative	34 (11.2)
Neither positive nor negative	145 (47.9)
Somewhat positive	94 (31.0)
Very positive	17 (5.6)
Types of received support (*n* = 303)	
Emotional support (e.g., demonstrations of acceptance, love)	155 (51.2)
Advice and recommendations to solve problems	142 (46.9)
Constructive criticism	110 (36.3)
Instrumental support (e.g., money, services)	37 (12.2)
Frequency of social support (*n* = 303; *M* = 2.27; SD = 1.0)	
Never	87 (28.7)
Seldom	86 (28.4)
Sometimes	96 (31.7)
Frequently	28 (9.2)
Always	6 (2.0)
Importance of social support (*n* = 216; *M* = 3.17; SD = 1.0)	
Not important	13 (4.3)
Slightly important	48 (15.8)
Indifferent	57 (18.8)
Important	86 (28.4)
Very important	12 (4.0)
Missing data	87 (28.7)
Discrimination experience (*n* = 303; *M* = 0.58; SD = 0.5)	
Yes	176 (58.1)
No	127 (41.9)
Reasons for being discriminated against (*n* = 303)	
Weight/body type	120 (39.6)
Sexual orientation	100 (33.3)
Beliefs (i.e., political leaning, religious beliefs)	67 (22.1)
Gender expression	46 (15.2)
Skin color	41 (13.5)
Nationality	41 (13.5)
Ethnicity	35 (11.6)
Having an STI	14 (4.6)
Catfishing experience (*n* = 303; *M* = 1.87; SD = 0.8)	
Yes	121 (39.9)
No	101 (33.3)
Does not know	81 (26.7)

Regarding social support, 28.7% (*n* = 87) of participants indicated that they had never received support from people met through apps, 28.4% (*n* = 86) reported that they had received it a few times, 31.7% (*n* = 96) sometimes, 9.2% (*n* = 28) a lot of times, and 2.0% (*n* = 6) always. When asked about the type of received support, 51.2% (*n* = 155) specified that they had received some kind of emotional support (e.g., demonstrations of acceptance, love, and empathy), 46.9% (*n* = 142) received advice and recommendations to solve problems, 36.3% (*n* = 110) indicated getting constructive criticism about a particular situation, and 12.2% (*n* = 37) received instrumental support such as money or services. When asked about the importance of the received support, 4.3% (*n* = 13) reported that it had not been at all important, 15.8% (*n* = 48) rated it as not being important, 18.8% (*n* = 57) indicated being indifferent about it, 28.4% (*n* = 86) rated it had been important, and 4.0% (*n* = 12) very important.

Regarding discrimination, 58.1% (*n* = 176) of participants reported having experienced some form of discrimination when using apps. Most of these respondents (*n* = 120, 39.6%) attributed weight or physical appearance as the reason behind the attack, followed by sexual orientation (*n* = 100, 33.0%), beliefs (i.e., political leaning, religious beliefs; *n* = 67, 22.1%), gender expression (*n* = 46, 15.2%), skin color (*n* = 41, 13.5%), nationality (*n* = 41, 13.5%), ethnicity (*n* = 35, 11.6%), and having a STI (*n* = 14, 4.6%). When asked about catfishing, a total of 121 (39.9%) participants reported knowing that someone created a fake profile under their name, 81 (26.7%) did not know if someone had done it, and the rest of the respondents (33.3%, *n* = 101) had never experienced this.

### Relationships Between Variables

We analyzed the relationships between sociodemographic variables and app-use related variables. Correlation analyses showed no relations between age and the analyzed variables. One-way ANOVAs showed significant differences in some of the variables based on relationship status and monthly income. We found that people in a stable cohabiting relationship (*M* = 4.96, SD = 2.32) had a higher number of fulfilled expectations compared to those in a non-cohabiting stable relationship (*M* = 4.61; SD = 1.80), followed by people not in a relationship (*M* = 4.12, SD = 1.80), *F*(2, 302) = 3.26, *p* = .04. Regarding monthly income, people who reported having a lower income had higher number of unfulfilled expectations (*M* = 1.76, SD = 1.68) than those with higher incomes (wage between 395 and 1182 USD; *M* = 1.28, SD = 1.50; More than 1183 USD, *M* = 1.10, SD = 1.36), *F*(2, 302) = 4.28, *p* = .015. Finally, people within the higher income group shared less characteristics in their profiles (*M* = 6.95, SD = 3.45) than the two other income groups (wage less than 394 USD; *M* = 7.70, SD = 3.44; wage between 395 and 1182 USD; *M* = 8.81, SD = 3.90), *F*(2, 302) = 5.30, *p* = .005.

We found statistically significant correlations between app-use related variables (Table 4); however, these correlations were very weak. Analyses revealed that having more apps correlated positively with the amount of time spent on them (*r* = 0.19, *p* < .001), the number of shared profile characteristics (*r* = 0.25, *p* < .001), the number of sought characteristics in other users’ profiles (*r* = 0.27, *p* < .001), and the number of fulfilled expectations (*r* = 0.13, *p* = .02). Also, having more apps correlated positively with the overall rating of the experience as app users (*r* = 0.11, *p* = .05), and being discriminated-against (*r* = 0.12, *p* = .04).

**Table 4:** Correlations between app use-related variables

		1	2	3	4	5	6	7	8	9	10	11
1	Number of used apps	1										
2	Spent time per day	.189**	1									
3	Number of fulfilled expectations	.133*	.145*	1								
4	Number of unfulfilled expectations	− .032	.018	− 0.32	1							
5	Number of shared characteristics	.248**	.086	.175**	.022	1						
6	Number of sought characteristics	.271**	.088	.220***	− .455**	.514**	1					
7	Overall experience rating	.114*	.110	.162**	.039	.282**	− .336**	1				
8	Frequency of social support	.073	− .002	.097	.069	.217**	− .234**	.255**	1			
9	Importance of social support	.112	− .010	.102	.073	.180**	− .169*	.332**	.349**	1		
10	Discrimination experience	.121*	.087	.111	.125*	.004	.136*	− .118*	− .008	.051	1	
11	Catfishing experience	− .095	− .111	− .071	− .072	− .158**	− .012	− .131*	− .083	− .133	− .056	1

*p < .05; **p < .01, two-tailed

The number of fulfilled expectations was positively correlated with the average time spent on apps (*r* = 0.15, *p* < .001), the overall rating of their experience (*r* = 0.28, *p* < .001), the frequency of social support (*r* = 0.22, *p* < .001), and the importance they give to the received support (*r* = 0.18, *p* < .001). Also, the number of fulfilled expectations was higher among those who share more characteristics in their profile (*r* = 0.18, *p* = .002) and those who seek more characteristics in other users’ profiles (*r* = 0.22, *p* < .001). In turn, the number of unfulfilled gratifications was negatively correlated with the overall rating of the quality of their app experience (*r* = − 0.34, *p* < .001), the frequency of support (*r* = − 0.23, *p* < .001), and the perceived importance of the received support (*r* = − 0.17, *p* = .01). Unfulfilled gratifications were also positively correlated with the number of discrimination experiences (*r* = 0.14, *p* = .02).

Higher quality of app experience was positively correlated with the frequency (*r* = 0.26, *p* < .001) and the importance attributed to the received support (*r* = 0.33, *p* < .001), but negatively correlated with the number of experiences of discrimination (*r* = − 0.12, *p* = .04) and catfishing (*r* = − 0.13, *p* = .23). Also, higher quality ratings were positively associated with number of profile self-presentation characteristics (*r* = 0.16, *p* = .005). Regarding social support, those who received it more frequently were more prone to perceive those expressions as highly important (*r* = 0.35, *p* < .001).

## Discussion

This exploratory study sought to describe several aspects of GSN app use in an online-collected sample of SMM residing in Ecuador. We believe it is important to generate efforts that allow scholars to better comprehend use patterns among SMM populations in parts of the world where research is still limited. Expanding research in Latin American countries could be useful to examine potential similarities across regions and determine whether cultural factors permeate SMM socialization experiences.

Homonegativity, religiosity, machismo, and conservatism may create a context in which GSN apps are perceived as safer environments than offline settings.However, it is also possible that these same cultural factors create a favorable context for hate practices against minorities to occur, as several reports of hate crimes seem to suggest (e.g., Diario El Comercio, 2020; Martínez, 2020). We hope that this research expands the growing body of literature produced in Latin America by focusing on variables that include usage and profile characteristics, expectations, experiences, social support, and discrimination. This information could be used to better understand, from a quantitative approach, SMM experiences on apps and their impact on their lives.

### Characteristics of App Use

Our results provide evidence that GSN apps are important socialization contexts for most SMM. Participants in our sample reported using between one to nine apps, with an average number of three apps, and up to 3 h of use per day. Grindr was the most frequently selected app, followed by Tinder, a non-SMM specific app, and other social networks like Facebook and Instagram. These results hold similarities to those reported by Queiroz et al. (2019) in Brazil who found Tinder and Grindr to be the most commonly used apps in their sample. According to these authors, Grindr has caused a significant impact on queer communities worldwide, defining a model for similar applications, and changing the way sexual minorities socialize by reducing access barriers and the likelihood of encountering homophobia.

Furthermore, in line with Chow et al.'s (2017) findings in Peru, we found that social media such as Facebook and Instagram were frequently used venues among SMM. In a context such as Ecuador’s, in which homophobia is frequent both in private and public spaces (INEC, 2013), social media may constitute safer spaces for some SMM to socialize and meet romantic partners without necessarily exposing themselves on widely known SMM-specific apps such as Grindr. Future research in the country should identify how non-SMM specific apps are being used as places to chat and meet other queer members. There may be specific characteristics among men who only use social media to connect with other SMM, compared to those who use both SMM-specific apps and other applications. As Gudelunas (2012) suggests, social networks are probably being used alongside SMM-specific apps to cross-reference information about potential partners.

### Sought and Fulfilled Gratifications

Sought gratifications could provide further insight into app use patterns among the men in the sample. According to our data, getting distracted, connecting with people with a similar sexual orientation, and having sexual encounters were the three most frequently endorsed gratifications. We are aware that these results might be explained by the fact that we explored expectations on different social networking apps, not only those for dating purposes. However, our findings suggest that GSN apps serve both sexual and non-sexual purposes. These findings are similar to those reported by Rice et al. (2012) who found that community building, entertainment, and socialization were the most important reasons for using apps. Nonetheless, we believe these results open new questionings about app use among SMM in the country. Expectations, especially those related to the development of connectedness to the queer community may be highly endorsed by men in parts of the country where physical access to queer spaces is limited or may represent physical or psychological risk. Considering that we analyzed the total number of fulfilled and unfulfilled expectations in the sample, future studies should examine specific expectations-such as those related to connectedness-based on age, ethnicity, socioeconomic status, and geographic location (e.g., rural areas).

Regarding fulfilled gratifications, the majority of the respondents felt that their initial expectations had been met when using apps; however, almost half of the participants reported that they had not been able to meet someone to have a stable and romantic relationship with. Due to the strong sexual nature of some apps, especially those directed specifically at SMM, some users may experience dissatisfaction and unmet expectations. Research has reported that apps can become hostile environments to those seeking romantic connections leading people seeking non-sexual relationships to be “judged, ignored or blocked after disclosing their relationship goals with others” (Zervoulis et al., 2020, p. 4). It is possible that not having those expectations met, ultimately affects people’s experiences in the apps and generate the ambivalent feelings expressed by the men in the sample regarding the quality of their experience.

### Shared and Sought Profile Characteristics

When asked about the characteristics they share and those that they seek in other peoples’ profiles, we found that participants’ age and photographs were the most common traits displayed on their profiles. Among those with profile pictures, most of them (44.2%, *n* = 134) indicated using a photograph that contained their faces. This finding is consistent with previous research that highlights that men usually disclose their faces in their profiles (Miller, 2015a). As Miller (2019) asserts, exposing the face could increase the potential of getting contacted, confirm the profile’s authenticity, or present to others as a visible and proud member of the gay community. Our results also reveal that profile pictures are important information taken to account when exploring other users’ profiles. In this study, we found that 77.6% (*n* = 235) of participants reported that photographs were commonly searched traits when viewing a person’s profile. This confirms observations on the importance of photographs when selecting users to talk and reply to (Miller, 2019).

Moreover, approximately 15% of participants reported using pictures different from their faces or bodies, including photographs of pets, logos, and fake pictures. These results may be attributed to feelings of internalized homonegativity among men in the country. Highly stigmatizing contexts can lead some men to develop feelings of guilt, shame, and lowered self-esteem as a result of the internalization of negative societal messages about homosexuality and bisexuality (Berg et al., 2016; Herek, 2009). To further explore this, future studies in the country could analyze differences in profile characteristics based on psychosocial variables (i.e., well-being, internalized homonegativity).

Sexual role was another important trait commonly shared and searched by users. Consistent with previous studies, displaying the sexual role may be a way to transmit an image of sexual dominance or submission to others, traits associated with rigid-gender roles and heteronormativity (Brooks et al., 2017; Moskowitz & Roloff, 2017). Research on the meanings of sexual roles in the gay community has revealed that labels such as being a “top” (i.e., the inserting partner), are usually associated with being “straight” and “masculine” whereas being the “bottom” (i.e., the anally receptive partner) is associated with being “gay” and “feminine” (Brooks et al., 2017; Johns et al., 2012; Moskowitz et al., 2008; Moskowitz & Roloff, 2017). We believe that this information is used, for some men, to convey an image aligned to the hegemonic masculinity prototype of *macho* and for others to search for partners to fulfill their sexual desires. Height, the fourth most looked up and shared trait may also be a part of these stereotyped ideas of attractiveness.

### User Experiences, Social Support, and Discrimination

Information on users’ experiences revealed that discrimination is common in the context of GSN apps. We found that approximately 60% (*n* = 181) of participants report having experienced some form of discrimination, indicating that the most common reasons for being discriminated against were weight and physical appearance. We also found a positive and significant correlation between time as an active GSN app user and higher experiences of discrimination. These results are salient because they add to the literature that highlights the existence of weight-related discriminatory practices and behaviors among the gay community (Conte, 2018; Miller, 2015a). This finding also supports the idea that GSN app usage may expose their users to factors associated with body-image disorders and body dissatisfaction (Filice et al., 2019).

We additionally found evidence of discrimination experiences based on other characteristics such as beliefs, skin color, ethnicity, nationality, and gender expression. In our sample, between 10 and 20% of participants reported being attacked based on these traits. These findings may reflect Ecuadorian idiosyncrasy. Historically, Ecuador has been a conservative, racist, classist, and *machista* society, marked by colonialist oppression practices against minorities (Ayala, 2002). As several authors have suggested, apps act as contexts where users reproduce privilege and power systems that position some SMM in hierarchical positions that differ based on the intersection of multiple identities (i.e., non-white, migrant, feminine SMM; Rubin & McClelland, 2015; Shield, 2018). Future studies would benefit from adopting intersectional approaches to examine how the convergence of different identity traits mark the experiences of SMM app users in the country.

Despite the existence of discrimination, our findings show that men also have positive experiences. When asked about social support, 60.0% (*n* = 182) of participants reported that they had received some sort of support either a few times or sometimes. Respondents suggested that the most common forms of support were either emotional (e.g., expressions of acceptance) or informational (e.g., advice) and to 32.4% these forms of support had been either important or very important. Correlation analyses further suggested that a higher number of used apps increased the likelihood of received support. In other words, using more apps could lead to higher access to sources of support.

### Relationship Between Variables

Finally, we did not identify significant relationships between app-use related variables and sociodemographic characteristics such as sexual orientation, ethnicity, nationality, education level, and religious practicing. Nevertheless, our findings suggest that participants in cohabiting relationships had a higher number of fulfilled expectations compared to those in non-cohabiting relationships and those who were single. Previous studies in the U.S. and Brazil have indicated that partnered men commonly use GSN apps as ways to satisfy sexual and social gratifications, and experience relational benefits and costs as a consequence of their app use (Goedel & Duncan, 2015; Macapagal et al., 2016; Queiroz et al., 2019). One explanation is that participants who reported being in cohabiting relationships were able to meet their romantic partners through apps, thus increasing the number of fulfilled expectations to the men in other groups. However, it is necessary to conduct further studies to reach more accurate conclusions. Also, we found that participants’ apps experiences vary based on their monthly income levels; SMM with lower earnings had more unfulfilled expectations than those with higher ones. Following previous statements on GSN apps as environments that reproduce power differences among members of the queer community (e.g., White, masculine, fit, rich; Miller, 2015a), men with lower incomes may have higher difficulties meeting expectations when interacting with men who seek certain characteristics in others.

### Limitations and Future Studies

This study had several limitations. First, the instrument employed was developed by the authors to gather information in Ecuador, a country in which no study on SMM’s GSN app use and experiences had been conducted. This situation may bring questions on the content validity of the instrument. To ensure that our instruments were sensitive to the context, we constructed a survey that was reviewed by four experts in the field of sexuality, sexual health, and gender issues. While we believe this helped us develop an instrument that gathered pertinent and useful information, we believe that future studies should revise some of its aspects. For example, we grouped social media such as Facebook and Instagram with other apps such as Grindr or Tinder. We believe it would be of great use for future studies to explore the participant’s perceptions and behaviors separately. This would help better identify the gratifications sought and met in SMM-specific apps versus other non-SMM specific apps and other social media.

Also, we used online snowball sampling methods to recruit our participants. To avoid invading people’s privacy, we decided not to create a profile inside each of the apps to promote the study. However, the use of this sampling method can have consequences in terms of the underrepresentation of certain sociodemographic groups (e.g., Black, Indigenous SMM) (Burrell et al., 2012; Sullivan et al., 2011). Thus, future studies could try to recruit app users directly. Also, despite not recognizing any potential cases, researchers should take precautions to avoid repeated entries during data collection.

Future studies should attempt to gather a bigger and more representative sample. In this study, we were able to recruit a sample comprised of mainly gay, single, cisgender, multiracial men in their mid-20’s. Despite this being probably the average characteristics of men using apps in the country, experiences of app users may vary widely according to their age, ethnicity, gender identity, sexual orientation, and relationship status. Future research should consider ways to locate more diverse populations and analyze their experiences using apps.

Despite its limitations, this research has allowed us to gather a broad perspective of GSN app use and its importance for SMM in a country where research is still limited. Future studies could examine possible differences in app-use related variables based on the main app used, type of app (i.e., SMM-specific vs. non-SMM specific), age group, ethnicity, religious affiliation, religiosity, and current relationship status. For example, expectations, support, and discrimination experiences may vary among younger and older cohorts, as well as those who are in a stable relationship, and those who are not. Larger samples, with higher heterogeneity in these variables, would allow making these comparisons.

Furthermore, we believe that research would benefit from comprehensive, context-situated, qualitative methods that analyze the different dynamics that occur among SMM who use apps in Ecuador. It would be of great value to examine how heteronormativity, homonegativity, hegemonic masculinity, and other cultural factors affect the interactions between men. Qualitative approaches would allow further comprehend the contrasting nature and effects of discriminatory and supportive behaviors inside apps.

Finally, future studies may analyze apps’ potential to increase users’ sexual networks. SMM are usually considered high populations for acquiring and transmitting HIV and other STIs (Holloway et al., 2014; Landovitz et al., 2013; Wei et al., 2019; Zou & Fan, 2017). To date, there is no current study on this matter in Ecuador, despite the high prevalence of HIV among these groups (UNAIDS, 2020). Future research must examine how popular socialization contexts such as GSN apps contribute to the exposure of SMM to these conditions and the set of safe-sex behaviors adopted to prevent them.

### Conclusions

GSN apps usage has widely grown throughout the world; Ecuador is certainly no exception. This study has revealed the usage and profile characteristics of these apps, users’ expectations, and positive and negative experiences in an online-recruited sample of SMM residing in the country. Results highlight the importance of closely examining the dual nature of apps. On the one hand, they can be contexts to safely socialize, share, and obtain support from other men, but, on the other, discriminatory practices can occur. Due to its exploratory nature, this study should be viewed as the starting point for future endeavors directed at examining different aspects of GSN apps use among commonly hidden populations.

## Data Availability

The data that support the findings of this study are available from the corresponding author upon reasonable request.
